# FAT10 protects against ischemia-induced ventricular arrhythmia by decreasing Nedd4-2/Nav1.5 complex formation

**DOI:** 10.1038/s41419-020-03290-3

**Published:** 2021-01-05

**Authors:** Xiao Liu, Jin Ge, Chen Chen, Yang Shen, Jinyan Xie, Xin Zhu, Menglu Liu, Jinzhu Hu, Leifeng Chen, Linjuan Guo, Qiongqiong Zhou, Xia Yan, Yuming Qiu, Rong Wan, Ali J. Marian, Kui Hong

**Affiliations:** 1 Department of Cardiovascular Medicine, The Second Affiliated Hospital of Nanchang University, 330006 Jiangxi, China; 2 Jiangxi Key Laboratory of Molecular Medicine, 330006 Jiangxi, China; 3 General Surgery Department, The Second Affiliated Hospital of Nanchang University, 330006 Nanchang, China; 4 Center for Cardiovascular Genetics at The University of Texas Health Science Center-Houston and Texas Heart Institute at St Luke’s Episcopal Hospital, Houston, TX 77030 USA

**Keywords:** Post-translational modifications, Arrhythmias

## Abstract

The human leukocyte antigen F-associated transcript 10 (FAT10) is a member of the small ubiquitin-like protein family that binds to its target proteins and subjects them to degradation by the ubiquitin–proteasome system (UPS). In the heart, FAT10 plays a cardioprotective role and affects predisposition to cardiac arrhythmias after myocardial ischemia (MI). However, whether and how FAT10 influences cardiac arrhythmias is unknown. We investigated the role of FAT10 in regulating the sodium channel Nav1.5, a major regulator of cardiac arrhythmias. *Fat10* was conditionally deleted in cardiac myocytes using *Myh6-Cre* and *Fat10*^*F/F*^ mice (*cFat10*^*−/−*^). Compared with their *wild-type* littermates, *cFat10*^*−/−*^ mice showed prolonged RR, PR, and corrected QT (QTc) intervals, were more likely to develop ventricular arrhythmia, and had increased mortality after MI. Patch-clamp studies showed that the peak Na^+^ current was reduced, and the late Na^+^ current was significantly augmented, resulting in a decreased action potential amplitude and delayed depolarization. Immunoblot and immunofluorescence analyses showed that the expression of the membrane protein Nav1.5 was decreased. Coimmunoprecipitation experiments demonstrated that FAT10 stabilized Nav1.5 expression by antagonizing Nav1.5 ubiquitination and degradation. Specifically, FAT10 bound to the lysine residues in the C-terminal fragments of Nav1.5 and decreased the binding of Nav1.5 to the Nedd4-2 protein, a ubiquitin E3 ligase, preventing degradation of the Nav1.5 protein. Collectively, our findings showed that deletion of the *Fat10* in cardiac myocytes led to increased cardiac arrhythmias and increased mortality after MI. Thus, FAT10 protects against ischemia-induced ventricular arrhythmia by binding to Nav1.5 and preventing its Neddylation and degradation by the UPS after MI.

## Introduction

Electrical remodeling of cardiac ion channels after myocardial ischemia (MI) plays a vital role in the development of ischemic arrhythmia, which is a major cause of sudden cardiac death^1^. Several cardiac ion channels, such as K^+^, Ca^2+^, and Na^+^ channels, participate in susceptibility to cardiac arrhythmias after MI. The main isoform of the voltage-gated Na^+^ channel Nav1.5 (a.k.a. *Scn5a*) encoded by the *Scn5a* gene is responsible for the generation of the Na^+^ current (*I*_Na_) and is the critical channel for maintaining normal cardiac rhythm^2^.

Nav1.5 remodeling provides a critical substrate for the generation of reentrant ventricular arrhythmias in border zones of the infarcted heart^3,4^. Post-translational modification of Nav1.5 is critical for regulating cardiac Na^+^ channels^5,6^. Nav1.5 is known to undergo Neddylation by Nedd4-2 (also known as Nedd4L), a ubiquitin E3 ligase, and subsequent degradation by the ubiquitin–proteasome system (UPS). The UPS plays a key role in regulating cardiac function in the ischemic heart^7,8^.

Ubiquitin-like proteins (UBLs) are a family of small proteins with structural and functional similarity to ubiquitin^9^ and are known to target ion channels. For example, a small ubiquitin-like modifier (SUMO) targets Kv4.2, thus affecting its biophysical properties and surface expression^10^. It also targets the current density of Cav1.2, which has been linked to sudden death and cardiomyopathy^11,12^. The human leukocyte antigen F-associated transcript 10 (FAT10) is a UBL that binds to proteins and tags them for proteasomal degradation. In addition to protein degradation, FAT10 is also involved in cell cycle regulation, immune response, and cell apoptosis^13^. We have shown that FAT10 antagonizes the ubiquitination of specific substrates and prevents their degradation^14^.

According to our findings, FAT10 protects cardiomyocytes against apoptosis in response to ischemic injury^15,16^. In the present study, we investigated the role of FAT10 in protecting against ischemic ventricular arrhythmia by binding to Nav1.5 and preventing its Neddylation and degradation by the UPS.

## Results

### The frequency and mortality of ventricular arrhythmia are increased in ischemic *cFat10*^*−/−*^ mice

**Fig. 1:**
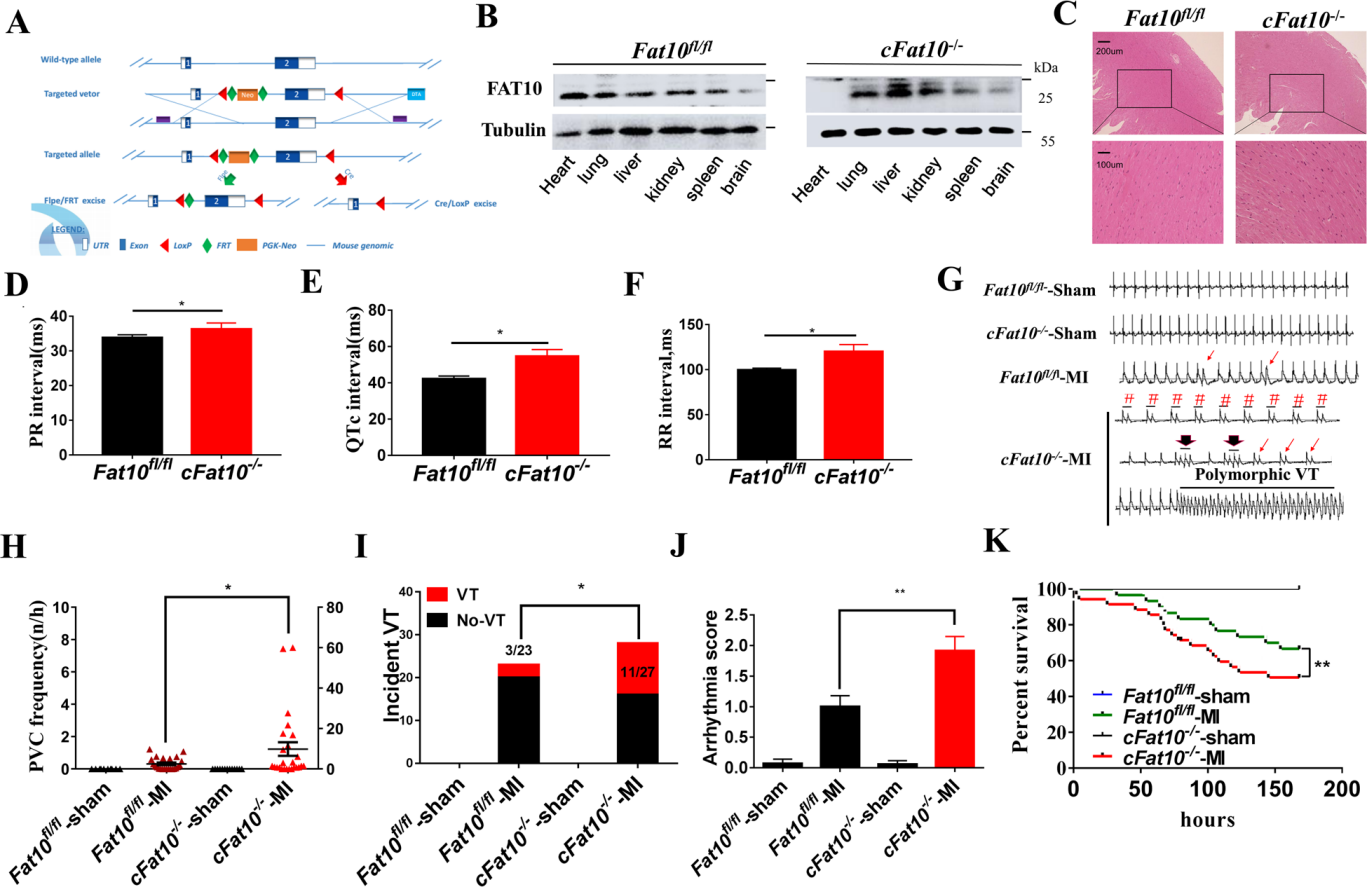
*Fat10*-deficient mice exhibit increased ventricular arrhythmia incidence and mortality rates after myocardial infarction (MI). **A** Schematic diagram of conditional cardiac *Fat10* knockout (*cFat10*^*−/−*^) generated by the homologous recombination technique. **B** Western blotting was performed to detect the expression of FAT10 in different tissues of *cFat10*^*−/−*^ mice and control littermates (*Fat10*^*fl/fl*^). **C** Hematoxylin–eosin staining of mouse heart sections from *cFat10*^*−/−*^ mice and *Fat10*^*fl/fl*^ mice. **D**–**F** Analysis of electrocardiograph (ECG) parameters from *cFat10*^*−/−*^ mice (*n* = 6) and *Fat10*^*fl/fl*^ mice (*n* = 10). PR interval (**D**); QTc interval (**E**); RR interval (**F**); (*p < 0.05). **G** Telemetric recordings of ECG tracings of ventricular arrhythmia in freely moving *Fat10*^*fl/fl*^-Sham, *cFat10*^*−/−*^-Sham, *Fat10*^*fl/fl*^-MI, and *cFat10*^*−/−*^-MI group mice (red arrows indicate PVCs, and black arrows indicate nonsustained ventricular tachycardia (VT), # indicates ventricular bigeminy). **H**–**J** Average paroxysmal PVCs (**H**), cumulative incidence of ventricular arrhythmias (**I**), and arrhythmia scores (**J**) of *Fat10*^*fl/fl*^ -MI group mice (*n* = 23) and *cFat10*^−^^*/−*^-MI group mice (*n* = 27) (*p < 0.05). **K** Kaplan–Meier curves were used to evaluate differences among the indicated groups of mice in terms of overall survival (**p < 0.01).

The FAT10 gene was deleted specifically in cardiac myocytes (*cFat10*^*−/−*^) upon insertion of a floxed allele encompassing exon 2 of the *Fat10* gene by homologous recombination and subsequent deletion of the floxed exon using myosin heavy-chain 6-Cre (*Myh6*-Cre) mice (Fig. 1A). Successful removal of the *Fat10* gene was confirmed by western blot (WB) and immunofluorescence (IF) analyses (Fig. 1B and Supplementary Fig. S1A). The *cFat10*^*−/−*^ mice displayed normal cardiac size and function, as assessed by echocardiography and histopathologic analysis, compared to those of their age- and sex-matched *wild-type* littermates (*Fat10*^*fl/fl*^) (Fig. 1C, Supplementary Fig. S1B, and Supplementary Table S1). Likewise, myocardial fibrosis was not increased in the *cFat10*^*−/−*^ mice, as assessed by Masson’s trichrome and Sirius red staining (Supplementary Fig. S1C–E).

Cardiac rhythm was assessed in conscious mice by telemetric cardiac rhythm monitoring, and no spontaneous arrhythmias were observed in the *cFat10*^*−/−*^ or *Fat10*^*fl/fl*^ mice. However, the PR (Fig. 1D), corrected QT (QTc) (Fig. 1E), RR (Fig. 1F), and QT intervals (Supplementary Fig. S1F) were significantly prolonged and the heart rates were significantly decreased (Supplementary Fig. S1G) in the *cFat10*^*−/−*^ mice compared to *Fat10*^*fl/fl*^ mice.

Considering the electrocardiographic (ECG) abnormalities, a mouse model of MI was established to assess the effect of *Fat10* gene deletion on ischemic cardiac arrhythmias^15^. Myocardial infarct size and cardiac function were similar in *Fat10* knockout mice and *Fat10*^*fl/fl*^ mice (Supplementary Fig. S1I–L). *Fat10* deletion was associated with a higher incidence of ventricular arrhythmia, as the *cFat10*^*−/−*^ mice post MI had a significantly higher prevalence of premature ventricular contractions (PVCs) (3.69 ± 0.33 vs. 0.43 ± 0.03 PVCs/h, respectively, *p* = 0.004) and nonsustained or sustained ventricular tachycardia episodes (0.39 ± 0.1 vs. 0.28 ± 0.01 episodes/h, *p* = 0.04) (Fig. 1G, H and Supplementary Fig. S1H). Specifically, 11 of the 27 (41%) *cFat10*^*−/−*^ mice showed an increased frequency of ventricular arrhythmias, whereas only 3 of the 23 (13%) *Fat10*^*fl/fl*^ mice post MI showed ventricular arrhythmia (*p* = 0.03; Fig. 1I). Moreover, *cFat10*^*−/−*^ mice showed a marked increase in the arrhythmia score (*p* = 0.003; Fig. 1J), as defined previously^17^, and a higher mortality rate than the *Fat10*^*fl/fl*^ mice (Fig. 1K).

### Nav1.5 expression in both the membrane and cytoplasm is reduced in ventricular myocytes isolated from *cFat10*^*−/−*^ mice

**Fig. 2:**
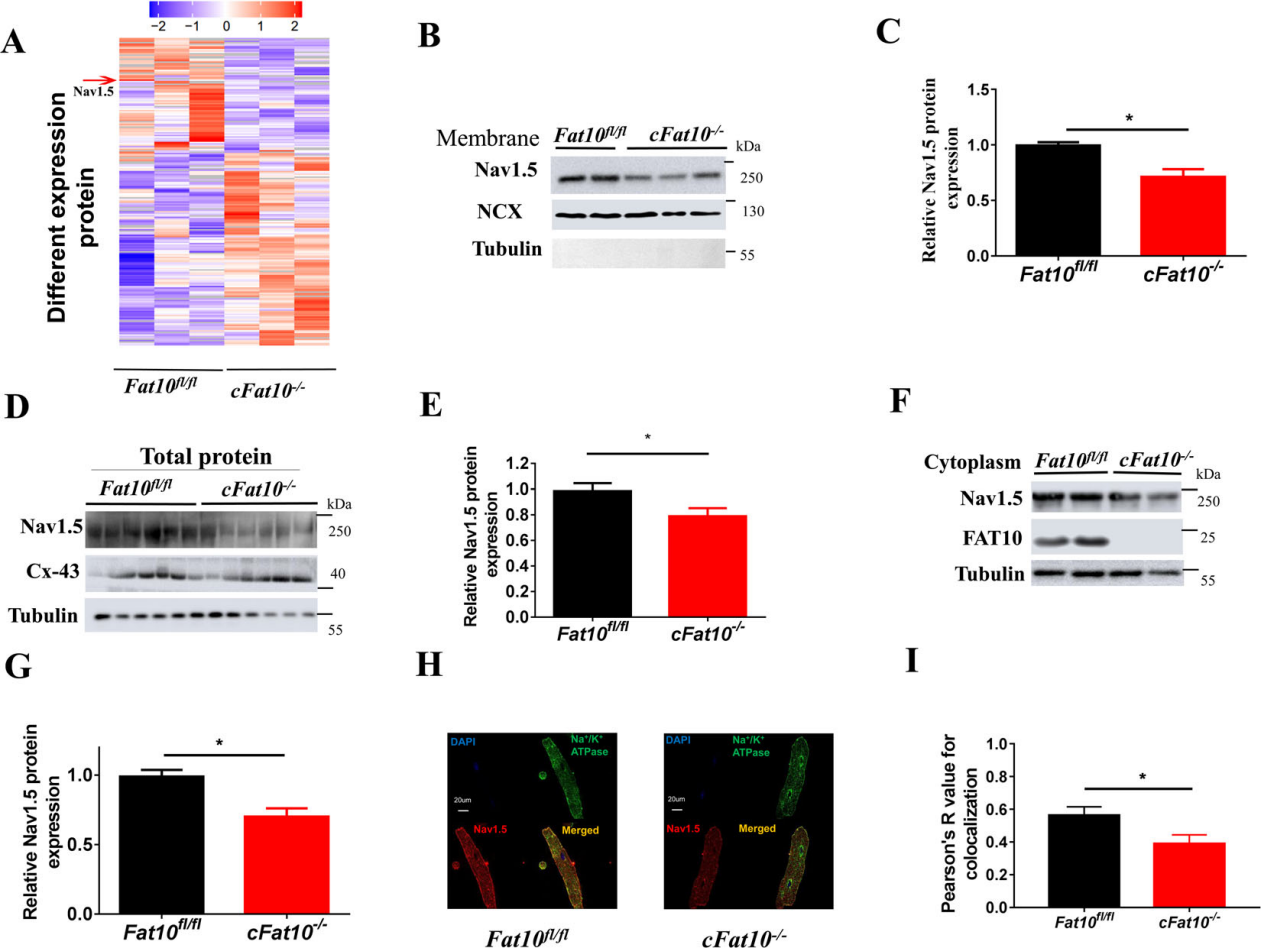
Nav1.5 protein expression is reduced in *F*at*10*-deficient ventricular myocytes. **A** Mass spectroscopic analysis was performed to detect protein expression in heart tissue samples from *Fat10*^*fl/fl*^ mice (*n* = 3) and *cFat10*^*−/−*^ mice (*n* = 3). Heatmap showing the differential expression of membrane proteins. **B**, **C** Representative immunoblots (**B**) and quantitative data (**C**) of membrane Nav1.5 protein expression in heart tissues from *Fat10*^*fl/fl*^ and *cFat10*^*−/−*^ mice (*p < 0.05); NCX: Na^+^/Ca^2+^ exchanger. **D**, **E** Representative immunoblots (**D**) and quantitative data (**E**) of total Nav1.5 protein expression in heart extracts from *cFat10*^*−/−*^ and *Fat10*^*fl/fl*^ mice as detected by WB (*p < 0.05). **F**, **G** Representative immunoblots (**F**) and quantitative data (**G**) of cytoplasmic Nav1.5 expression in heart tissues from *cFat10*^−^^*/−*^ and *Fat10*^*fl/fl*^ mice (*p < 0.05) as detected by WB. **H**, **I** Representative immunostaining showing the degree of colocalization between Nav1.5 and Na^+^/K^+^ ATPase (**H**) and Pearson’s correlation coefficient for colocalization (**I**) in isolated cardiomyocytes (scale bar = 20 μm). All results are expressed as the mean ± SEM of independent experiments.

Analysis of the proteomics data from three *cFat10*^*−/−*^ and control mouse hearts showed that membrane Nav1.5 expression was significantly reduced in the *cFat10*^*−/−*^ heart (Fig. 2A), which was confirmed by WB (Fig. 2B, C). However, there was no significant difference in the expression levels of the Nav1.5 trafficking proteins SAP97 and MOG1 (Supplementary Fig. S2A) or in the *Scn5a* transcript levels between *cFat10*^*−/−*^ and *Fat10*^*fl/fl*^ mice (Supplementary Fig. S2B), while the Nav1.5 protein levels were reduced in both the cytoplasmic subfractions and the whole-heart protein extracts (Fig. 2D–G). Unlike Nav1.5, the expression of Connexin-43, Cav1.2, and Na^+^/K^+^ ATPase was not changed in the hearts of *cFat10*^*−/−*^ mice, as detected by immunohistochemistry and WB (Fig. 2D and Supplementary Fig. S2C, D). Consistent with these findings, IF staining of isolated cardiac myocytes demonstrated reduced expression and membrane localization of Nav1.5 as determined by Pearson’s correlation coefficient in cardiac myocytes isolated from the *cFat10*^*−/−*^ mice (Fig. 2H, I). These results suggested that decreased Nav1.5 expression is a putative mechanism for the increased incidence of ventricular arrhythmia after MI in *cFat10*^*−/−*^ mice.

### The electrophysiological function of Nav1.5 is reduced in *cFat10*^*−/−*^ cardiomyocytes

**Fig. 3:**
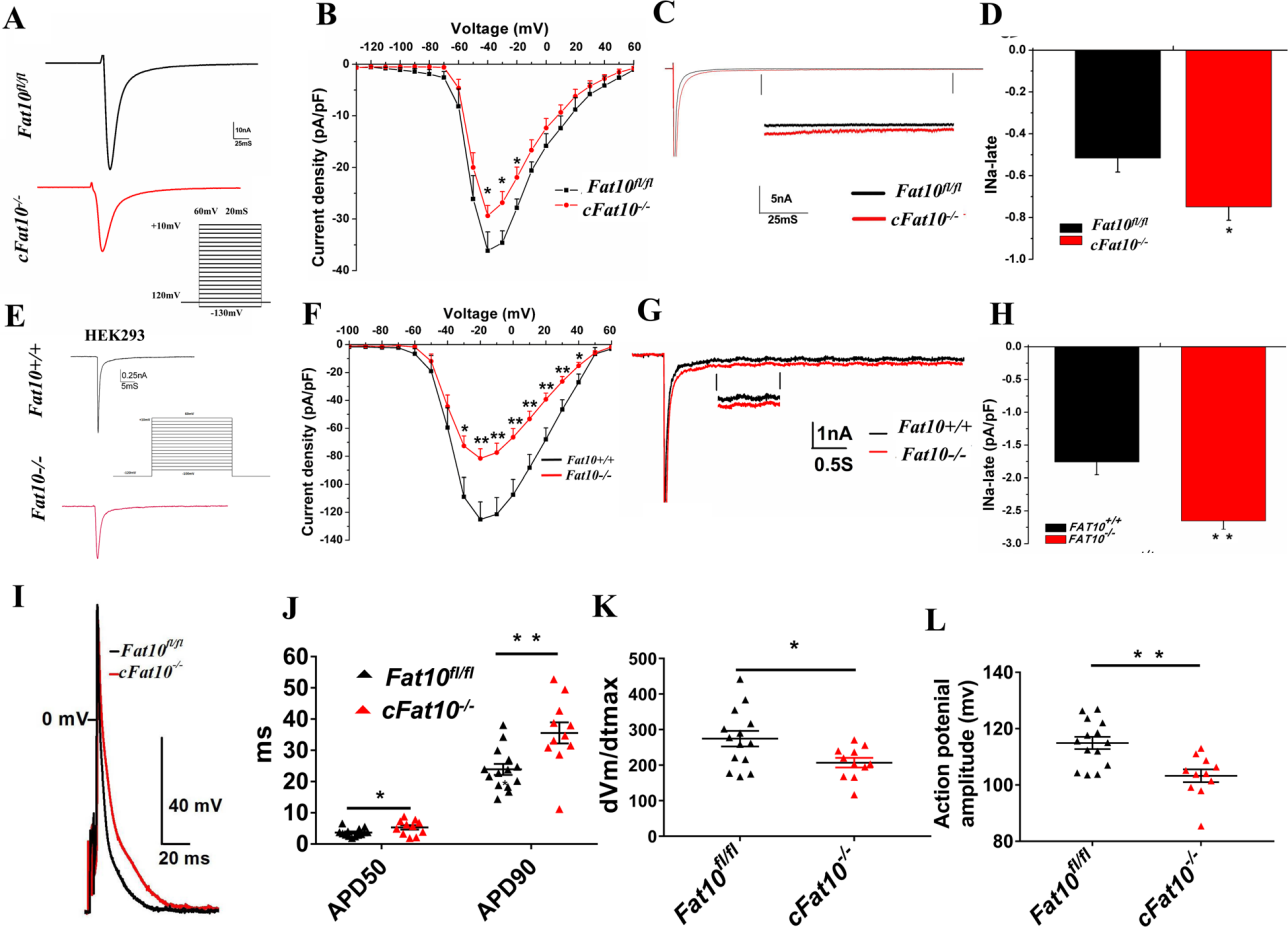
Sodium current (*I*_Na_) and action potentials (AP) of *cFat10*^*−/−*^ cardiomyocytes. **A**, **B** Peak *I*_Na_ in ventricular myocytes isolated from *cFat10*^*−/−*^ and *Fat10*^*fl/fl*^ mice. Representative traces of *I*_Na_ with a pulse protocol (**A**) and the current–voltage relationship of *I*_Na_ (**B**) are shown (*n* = 13 for *Fat10*^*fl/fl*^ cells and *n* = 15 for *cFat10*^*−/−*^ cells). **C**, **D** Late sodium current (*I*_Na,L_) was measured in response to 200 ms voltage steps to −20 mV from a holding potential of −120 mV (mean *I*_Na,L_ at 150 ms, *p < 0.05, *Fat10*^*fl/fl*^ *vs. cFat10*^*−/−*^). **E**, **F** *I*_Na,L_ in *wild-type* (*Fat10*^*+/+*^) HEK293 cells (*n* = 11) or *Fat10*^*−/−*^ HEK293 cells (*n* = 18) expressing *wild-type Scn5a*. Representative traces of *I*_Na,L_ with a pulse protocol (**E**) and the current–voltage relationship of *I*_Na_ (**F**). (*p < 0.05, **p < 0.01, *Fat10*^*+/+*^ vs. *Fat10*^*−/−*^ HEK293 cells). **G**, **H** *I*_Na,L_ was measured in response to 200 ms voltage steps to −20 mV from a holding potential of −120 mV (mean *I*_Na,L_ at 150 ms, *p < 0.05, *Fat10*^*+/+*^ vs. *Fat10*^*−/−*^ HEK293 cells). **I** Representative APs were recorded from *Fat10*^*fl/fl*^ (*n* = 14) and *cFat10*^*−/−*^ (*n* = 11) ventricular myocytes. **J**–**L** AP duration (**J**), velocity of the AP upstroke (**K**), and AP amplitude (**L**) in ventricular myocytes isolated from *cFat10*^*−/−*^ mice or *Fat10*^*fl/fl*^ mice (*p < 0.05, **p < 0.01).

To determine the functional effect of reduced Nav1.5 levels, the whole-cell patch-clamp technique was used to record the *I*_Na_ currents. The peak *I*_Na_ density was significantly reduced in the *cFat10*^*−/−*^ ventricular myocytes (Fig. 3A, B) (−38.7 ± 2.9 vs. −29.4 ± 2.0 pA/pF, *p* = 0.01), while changes in steady-state activation and inactivation were not observed (Supplementary Fig. S3A, B). However, the late *I*_Na_ (*I*_Na,L_) current was significantly increased in the *cFat10*^*−/−*^ myocytes (*p* = 0.02) (Fig. 3C, D).

To further corroborate these findings, a heterologous expression system was used to generate *Fat10* knockout HEK293 (*Fat10*^*−/−*^) cells by transfection with *CRISPR-Cas9* plasmids^14^. Compared to that in *wild-type* (*Fat10*^*+/+*^) cells, the peak *I*_Na_ current was moderately but significantly decreased in *Fat10*^−/−^ cells (−125.1 ± 12.5 vs. −81.6 ± 6.9 pA/pF; *p* = 0.003) (Fig. 3E, F). However, *I*_Na,L_ was significantly increased in the *Fat10*^*−/−*^ cells (*p* = 0.003) (Fig. 3G, H). There were no changes in the steady-state active or steady-state inactive relationships (Supplementary Fig. S3C, D). The peak *I*_Na_ current was significantly increased (−128.7 ± 12.1 vs. −191.2 ± 25.1 pA/pF, *p* = 0.02) in cells transfected with the *Flag-Fat10* plasmid (Supplementary Fig. S3E, F), whereas the *I*_Na,L_ was decreased (*p* = 0.02) (Supplementary Fig. S3G, H). No changes in the steady-state active or steady-state inactive relationships were observed (Supplementary Fig. S3I, J).

Action potential duration (APD) was significantly prolonged in the *cFat10*^*−/−*^ ventricular cardiomyocytes (Fig. 3I, J) (APD50: *p* = 0.03; APD90: *p* = 0.003). Moreover, the maximum and upstroke velocity (*p* = 0.02) and amplitude of AP (*p* = 0.001) were decreased (Fig. 3K, L) in the *cFat10*^*−/−*^ cardiomyocytes, while the resting membrane potential was not changed (*p* = 0.09, Supplementary Fig. S4). These AP changes in the *cFat10*^*−/−*^ cardiomyocytes were consistent with the ECG) phenotype (prolonged QTc and RR intervals), and these findings collectively suggested that *Fat10* deletion prolongs APD because of decreased *I*_Na_ and Na^+^ channel activity.

### FAT10 stabilizes Nav1.5 expression in cardiomyocytes by inhibiting its ubiquitination

**Fig. 4:**
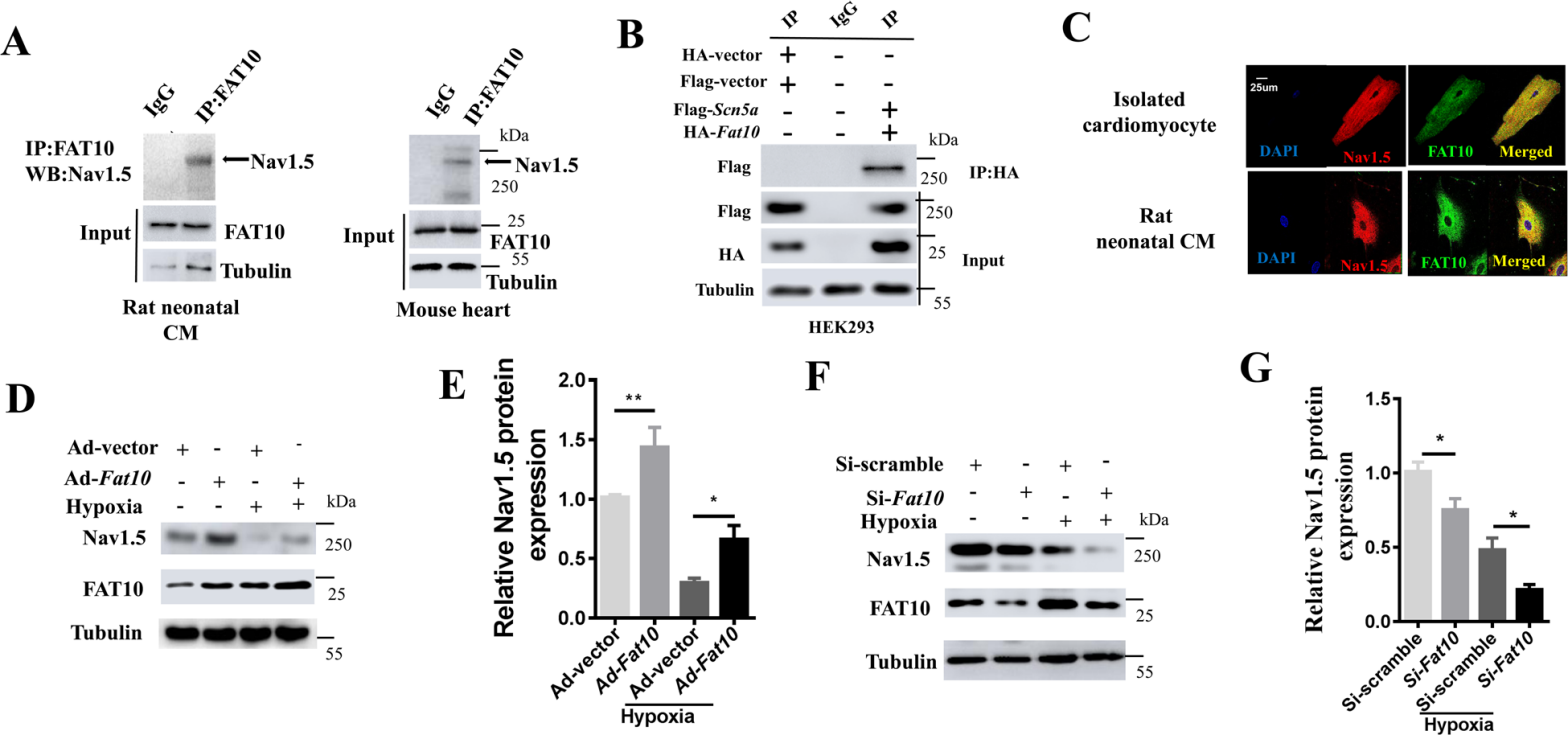
FAT10 regulates Nav1.5 protein expression in cardiomyocytes. **A** Coimmunoprecipitation (Co-IP) was performed to elucidate the association between FAT10 and Nav1.5 in neonatal rat cardiomyocytes (NRCMs) (left) and mouse hearts (right). **B** The association between FAT10 and Nav1.5 in HEK293 cells expressing Flag-Nav1.5 and HA-FAT10 was detected by Co-IP. **C** Photomicrographs showing the colocalization of FAT10 and Nav1.5 in cardiomyocytes (upper) and NRCMs (lower). **D**, **E** Effect of FAT10 overexpression on Nav1.5 expression levels in NRCMs under normoxic and hypoxic conditions as evaluated by WB (*p < 0.05, **p < 0.01). **F**, **G** Effect of Fat10 knockdown on the Nav1.5 expression levels in NRCMs under normoxic and hypoxic conditions as evaluated by WB (*p < 0.05, **p < 0.01). All results are expressed as the mean ± SEM of independent experiments.

**Fig. 5:**
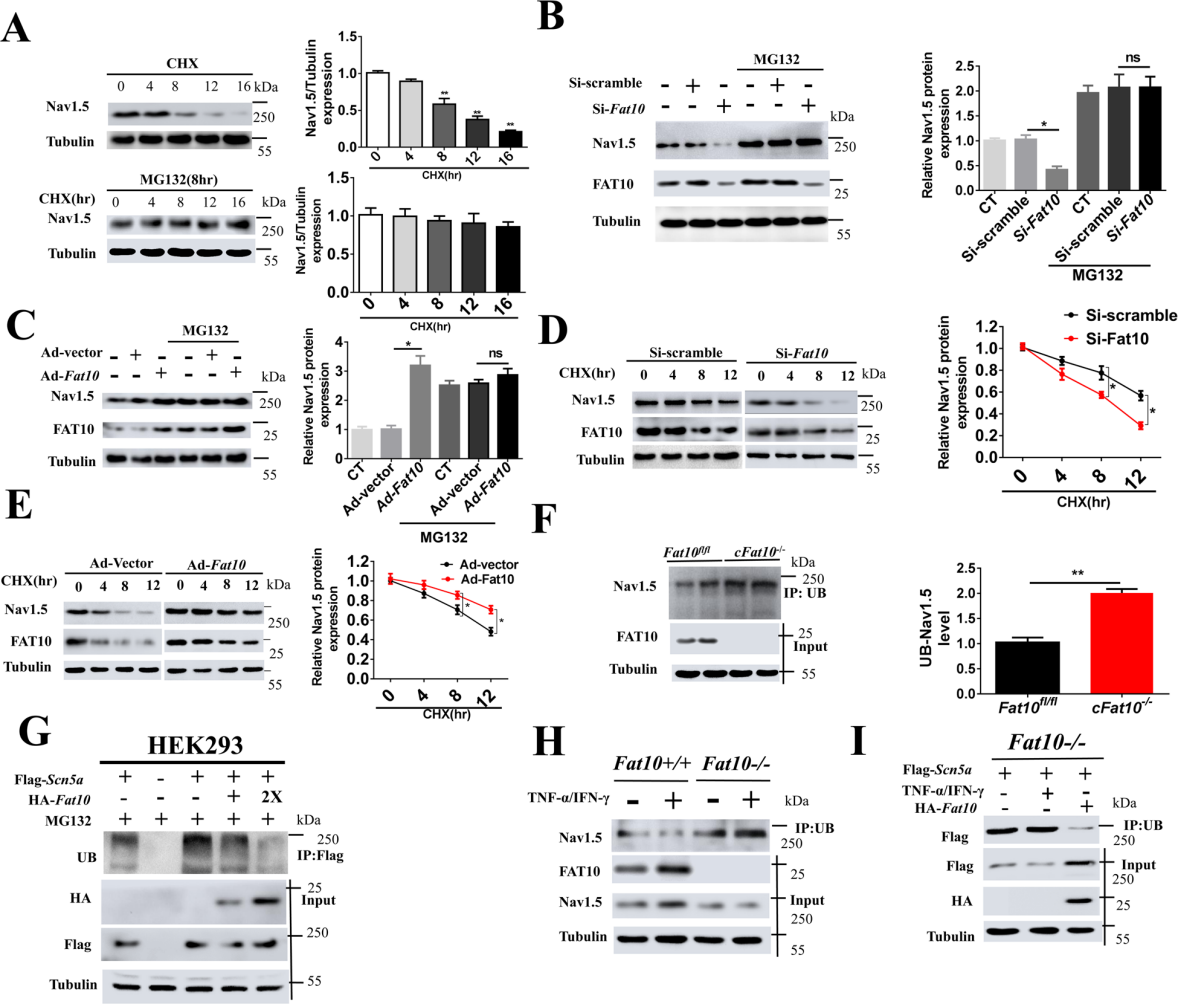
FAT10 stabilizes Nav1.5 expression in cardiomyocytes by inhibiting ubiquitination. **A** Representative immunoblots (left) and quantitative assessment of the protein (right) levels of Nav1.5 in NRCMs treated with cycloheximide (CHX, 20 μM) or MG132 (15 μM) for the indicated times. *p < 0.05 and **p < 0.01 vs. treatment in the 0 h group. **B**, **C** Representative immunoblots or quantitative data of Nav1.5 expression in NRCMs after adenoviral knockdown (**B**) or overexpression (**C**) of *Fat10*. Cells were treated with or without MG132 (*p < 0.05). **D** Western blotting was performed to detect the expression of FAT10 and Nav1.5 in NRCMs with or without FAT10 knockdown (*p < 0.05) treated with CHX. **E** Western blotting was performed to detect the expression of FAT10 and Nav1.5 in NRCMs with or without FAT10 overexpression treated with CHX (*p < 0.05). **F** Representative immunoblots (left) and quantitative data (right) of Nav1.5 ubiquitination levels after immunoprecipitation of Nav1.5 from cultured cardiomyocytes isolated after MG132 treatment for 8 h (*p < 0.05). **G** Representative immunoblots of the Nav1.5 ubiquitination levels in HEK293 cells transfected with the indicated plasmid DNA. Cells in each group were treated with MG132. **H** Representative immunoblots of the Nav1.5 ubiquitination levels in *Fat10*^*+/+*^ and *Fat10*^*−/−*^ HEK293 cells treated with or without TNF-α/IFN-γ. **I** Representative immunoblots of the Nav1.5 ubiquitination levels in the indicated groups of *Fat10*^*−/−*^ HEK293 cells. All results are expressed as the mean ± SEM of independent experiments.

To determine the mechanisms by which FAT10 deficiency affects Nav1.5 function, endogenous physical interactions of Nav1.5 and FAT10 were detected by coimmunoprecipitation (Co-IP) in neonatal rat cardiomyocytes (NRCMs) and adult cardiac tissue extracts (Fig. 4A and Supplementary Fig. S5A). The interaction was confirmed in HEK293 cells transfected with *Flag*-tagged *Ssc5a* and *HA*-tagged *Fat10* plasmid DNAs (Fig. 4B). Confocal microscopy showed colocalization of FAT10 and Nav1.5 in adult mouse myocytes and NRCMs (Fig. 4C). In NRCMs under normoxic and hypoxic conditions, overexpression of FAT10 was associated with increased levels of Nav1.5 protein expression (Fig. 4D, E), whereas downregulation of FAT10 had the opposite effect (Fig. 4F, G), and no changes in the mRNA levels were observed (Supplementary Fig. S5C). Interestingly, FAT10 protein expression was increased by hypoxia (Supplementary Fig. S5D, E). Moreover, Nav1.5 expression was also upregulated by endogenous FAT10 overexpression (induced by interferon-γ/tumor necrosis factor-α (IFN-γ/TNF-α)^18^) in NRCMs (Supplementary Fig. S5F). FAT10 affects the expression of its substrates by regulating the level of their ubiquitination^14^. Given that Nav1.5 is known to be degraded by ubiquitination, we analyzed the regulation of Nav1.5 by FAT10 through ubiquitination. Therefore, the Nav1.5 protein levels were assessed after treatment with cycloheximide (CHX) in the absence or presence of the MG132 proteasome inhibitor. As shown in Fig. 5A (upper), treatment with CHX significantly decreased the endogenous Nav1.5 protein expression in NRCMs in a time-dependent manner, which was nulled by the presence of MG132 (Fig. 5A, lower). Thus, these results indicate that the Nav1.5 protein undergoes degradation via the UPS pathway.

In a complementary set of studies, the effects of FAT10 on the expression of Nav1.5 in NRCMs were shown to be negated by inhibition of the UPS with MG132 (Supplementary Fig. 5B, C). Regardless of MG132 treatment, knockdown or overexpression of FAT10 in NRCMs was associated with an increased or reduced degradation rate of the Nav1.5 protein, respectively (Fig. 5D, E). In vitro experiments showed that the Nav1.5 ubiquitination levels were significantly increased in *cFat10*^*−/−*^ cardiomyocytes (Fig. 5F). We then performed an in vitro ubiquitination assay in HEK293 cells upon overexpression of Nav1.5 and FAT10, revealing that FAT10 overexpression decreased the levels of ubiquitinated Nav1.5 (Fig. 5G). Furthermore, treatment with IFN-γ/TNF-α was associated with reduced binding of Nav1.5 to ubiquitin in *Fat10*^*+/+*^ cells, but not in *Fat10*^*−/*^^*−*^ cells (Fig. 5H). In addition, in the *Fat10*^*−/−*^ cells, the binding of Nav1.5 to ubiquitin was rescued upon expression of *HA-Fat10* using plasmids, but not upon treatment with IFN-γ/TNF-α (Fig. 5I). Overall, these results show that FAT10 stabilizes Nav1.5 expression in cardiomyocytes by inhibiting ubiquitination.

### FAT10 antagonizes Nav1.5 ubiquitination through the C-terminal domain by decreasing the formation of the Nedd4-2–Nav1.5 complex

**Fig. 6:**
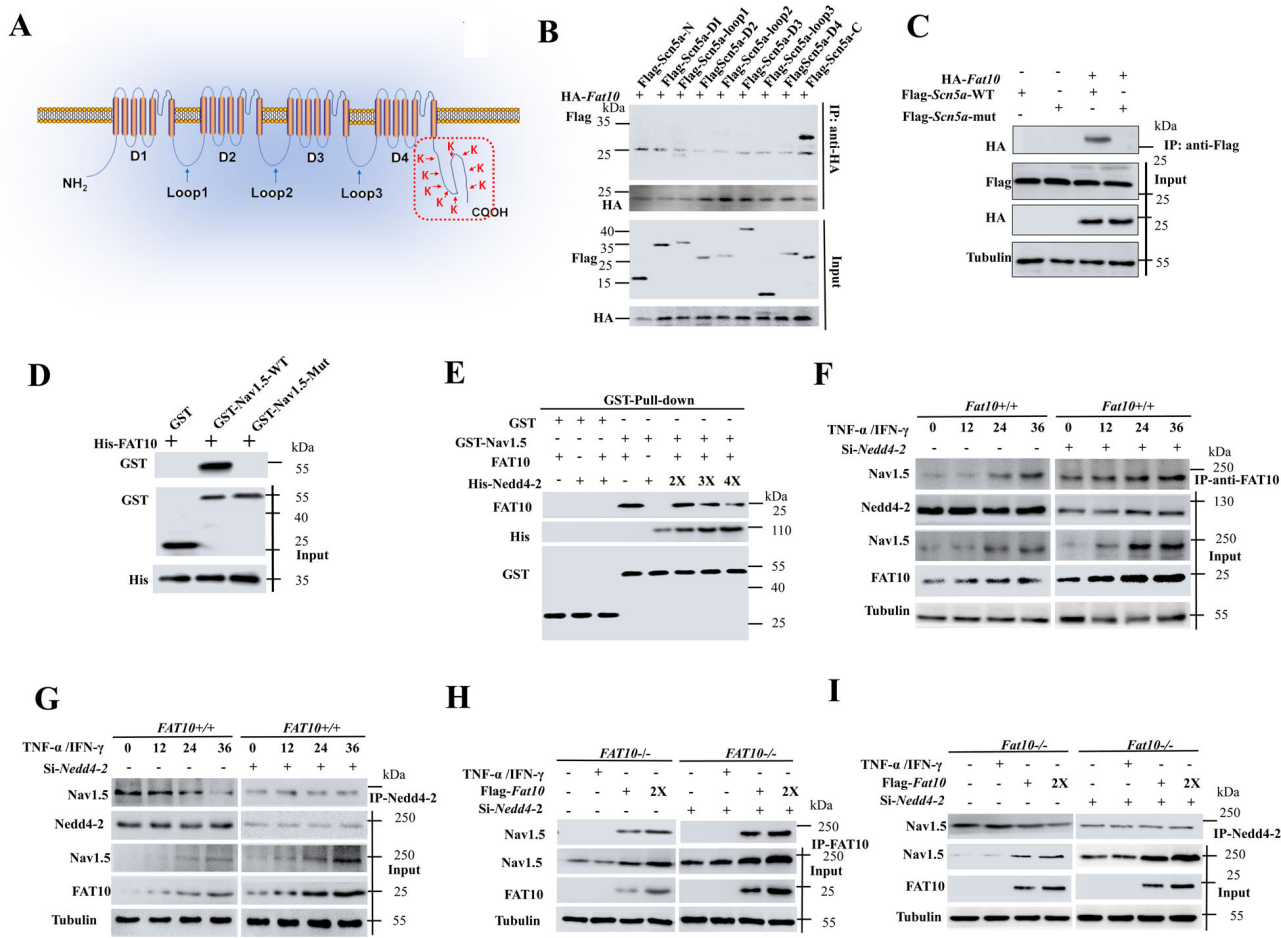
FAT10 antagonizes Nav1.5 ubiquitination by competing with Nedd4-2. **A** Schematic diagram of Nav1.5 deletions and mutants. **B** HEK293 cells were transfected with the indicated constructs and then lysed for the Co-IP assay using anti-Flag beads to detect HA-FAT10 binding. **C** Representative immunoblots of FAT10 binding with Nav1.5 in HEK293 cells co-transfected with *HA-Fat10* and *Flag-Scn5a wild-type* or the *Flag-Scn5a* mutation plasmid as detected by Co-IP. **D** GST pull-down experiments were performed to assess the association between FAT10 and Nav1.5. **E** Competitive binding of Nav1.5 was analyzed in a GST pull-down experiment. **F**, **G** *Fat10*^*+/+*^ HEK293 cells were transfected with *Si-Nedd4-2* or *Si-scramble* and treated with IFN-γ/TNF-α for the indicated times. Co-IP was performed to detect Nedd4-2–Nav1.5 and FAT10–Nav1.5 complex formation. **H**, **I** *Fat10*^*−/−*^ HEK293 cells were transfected with *Si-Nedd4-2/Si-scramble* and the *Flag-Fat10* plasmid. Co-IP was performed to detect Nedd4-2–Nav1.5 and FAT10–Nav1.5 complex formation. HEK293 cells were treated with IFN-γ/TNF-α to induce FAT10 expression. All results are expressed as the mean ± SEM of independent experiments.

To identify the Nav1.5 domain that binds to the FAT10 protein, nine fragments of the *Scn5a* (Nav1.5) gene were generated, as depicted in Fig. 6A, expressed in HEK293 cells and analyzed for their ability to bind FAT10. Co-IP studies showed that the C-terminal fragment (*Scn5a-C*), comprising residues 1978–2016, contained the FAT10 interaction domain (Fig. 6B). Therefore, a glutathione *S*-transferase (*GST*) pull-down assay performed using a *GST*-Nav1.5*-C* fusion protein (*GST*-Nav1.5) showed a direct interaction with FAT10 (Fig. S6A). Because FAT10 modifies its substrates by binding to the lysine sites on substrates^14^, a plasmid construct expressing a mutant *Scn5a*-C-terminal (*GST-Scn5a-*Mut) was constructed by mutating nine lysine residues to arginine. Interestingly, the introduction of the mutations abolished the binding of FAT10 to Nav1.5 in the Co-IP and *GST* pull-down assays (Fig. 6C, D). These findings suggested that FAT10 affects Nav1.5 expression by directly interacting with the lysine of *Scn5a-*C.

Nedd4-2 is known to interact with Nav1.5 to degrade Nav1.5 expression^19^, and we postulated that FAT10 affects Nav1.5 ubiquitination by altering the Neddylation of the Nav1.5 protein. Therefore, the binding of Nav1.5 to FAT10 and Nedd4-2 was analyzed under competitive conditions. Co-IP and WB experiments showed that overexpression of Nedd4-2 decreased the FAT10–Nav1.5 complex level in HEK293 cells (Fig. 6E), while overexpression of FAT10 decreased the Nedd4-2–Nav1.5 complex level (Supplementary Fig. S6B). Moreover, induction of FAT10 (IFN-γ/TNF-α) increased Nav1.5 expression and FAT10–Nav1.5 complex levels in a dose-dependent manner, while knockdown of Nedd4-2 did not alter the Nedd4-2–Nav1.5 complex level (Fig. 6F, G). Furthermore, the treatment decreased Nav1.5–Nedd4-2 complex levels, which was abrogated by knockdown of Nedd4-2 (Fig. 6G). Moreover, in *Fat10*^*−/−*^ cells, FAT10–Nav1.5 complexes were undetectable, whereas the Nedd4-2–Nav1.5 complexes were unchanged (Fig. 6H, I). Furthermore, when FAT10 expression was rescued by transfection with *Flag-Fat10* plasmid DNA, FAT10–Nav1.5 complex expression was increased, while the Nedd4-2–Nav1.5 complex level was unaltered by low Nedd4-2 expression (Fig. 6H, I).

**Fig. 7:**
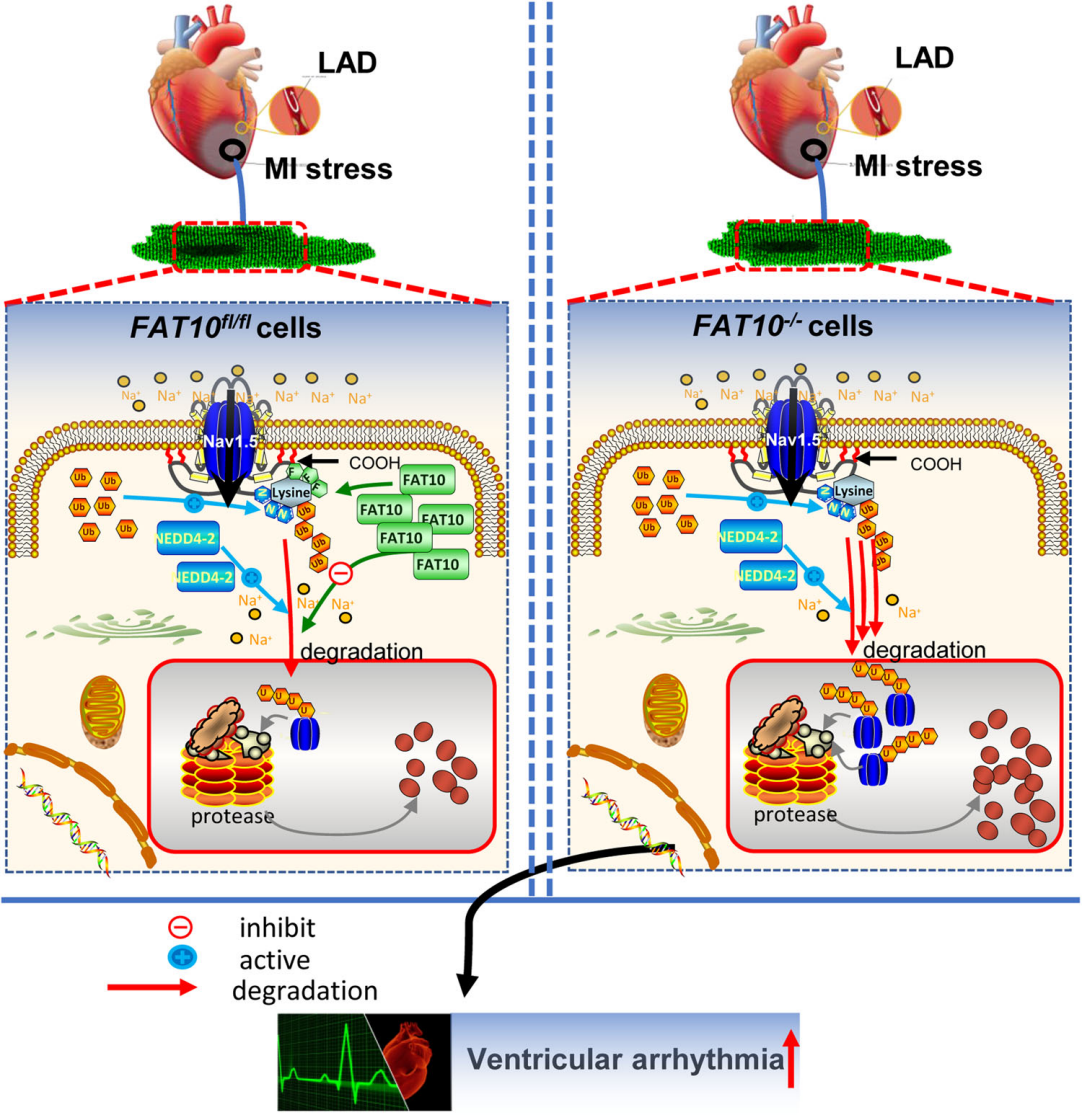
Working diagram. The graphical diagram illustrated that FAT10 protected against ischemia-induced ventricular arrhythmia by decreasing Nedd4-2–Nav1.5 complex formation.

Thus, altogether these data reveal that FAT10 stabilizes Nav1.5 expression, by decreasing the Neddylation of Nav1.5 and modulating Nav1.5 ubiquitination, thus protecting against ischemia-induced ventricular arrhythmia (Fig. 7).

## Discussion

FAT10 is a relatively newly discovered member of the UBL family. We previously reported that FAT10 protects cardiomyocytes against apoptosis, which is required to maintain cardiac function post-7 day MI^15,16^. In the present study, we identified an important role of FAT10 in ischemia-induced ventricular arrhythmia. We showed that FAT10 bound to the C-terminal fragments of Nav1.5, stabilizing Nav1.5 expression upon prevention of its Neddylation and degradation, ultimately increasing the *I*_Na_ current and AP in cardiomyocytes.

Recently, several ion channels, including kv7.1 and Nav1.2, have been shown to be modified by UBLs via processes such as SUMOylation^20^. The function of Nav1.5 is critical for cardiac excitability and electrical conduction^21^, and ubiquitination of Nav1.5 is an important mediator of Nav1.5 trafficking, internalization, and degradation^19,22^. However, little is known about whether Nav1.5 is modulated by UBLs. The findings of the present study provide the first evidence of Nav1.5 modification by FAT10 in cardiomyocytes. Ubiquitination and degradation of Nav1.5 were first reported in 2004^19^, and several ubiquitin E3 ligases have been implicated in the regulation of Nav1.5 ubiquitination^19,23^. For example, Nedd4-2 has been shown to regulate Nav1.5 ubiquitination by the PY motif in the C-terminal region, suggesting an important role of the C-terminal region in Nav1.5 ubiquitination. Consistent with previous studies, we showed that FAT10 regulated ubiquitination by interacting with the C-terminal domain of Nav1.5, exploiting lysine residues, as changing these residues abolished the interaction of Nav1.5 with FAT10. The findings illustrate that FAT10 prevents Neddylation and UPS-mediated degradation of Nav1.5.

Interestingly, we found that FAT10 expression was increased, while that of Nav1.5 was decreased under hypoxic conditions, and several factors may underlie this result. First, the degradation of Nav1.5 is regulated by complex molecular mechanisms, such as ubiquitination^5^ and autophagy^24^. In the present study, FAT10 overexpression stabilized the Nav1.5 protein by regulating its ubiquitination. The FAT10 protein is not unique in regards to the regulation of Nav1.5 ubiquitination, as several proteins have been demonstrated to participate in this process, such as UBC9^25^, UBR3, and UBR6^26^. Furthermore, under ischemia or hypoxia, the UBS and autophagic activities were increased^7,27^, which was potentially responsible for the decreased Nav1.5 protein expression^5,24^ However, the degradation of Nav1.5 could partially but not totally be rescued by the increased FAT10 expression under the conduction of ischemia by antagonizing Nav1.5 ubiquitination, demonstrating a cardioprotective effect of FAT10. These results might explain the increased FAT10 expression and decreased Nav1.5 expression under ischemic/hypoxia conditions.

Reduced expression of Nav1.5 decreases peak *I*_Na_, which results in progressive cardiac conduction defects with the widening of the QT interval^21^. In the present study, *Fat10* knockout was associated with reduced sodium channel activity because of decreased Nav1.5 levels, while steady-state activation and inactivation were not affected. The C terminus of Nav1.5 is lysine-rich and is one of the most conserved regions of the protein. Mutations or deletions involving lysine in the C-terminal domain may lead to abnormal Nav1.5 function and severe human arrhythmias. Relevant to these findings, a deletion mutation affecting lysine-1872 (located in the C terminus) has been reported in a large family with Brugada syndrome, a disease manifesting as decreased peak *I*_Na_^28^. The findings demonstrated that the C terminus might play an important role in regulating *I*_Na_ through the UPS pathway. Previous studies support the involvement of the C-terminal (Ser-Ile-Val [SIV]) domain of Nav1.5 in UPS-mediated degradation^29^. Another notable finding was increased *I*_Na,L_ in the *cFat10*^−^^*/−*^ cardiomyocytes, which may be attributed to reduced expression of the caveolin-3 (Cav3) protein, as reported previously^15^. Decreased Cav3 expression has been shown to increase the S-nitrosylation of Nav1.5, resulting in increased *I*_Na,L_ in cardiomyocytes^30^.

The findings of prolonged PR, RR, and QT intervals on ECG suggested slow conduction in the *cFat10*^*−/−*^ hearts, which may stem from altered tissue architecture, cell-to-cell coupling, and/or cardiomyocyte excitability^31–33^. In the present study, Nav1.5 expression was reduced, as were *I*_Na_, AP upstroke velocity (d*V*/d*t*max, a measure of sodium channel availability), and AP amplitude, which might explain the slow conduction velocity^34,35^. Moreover, the QTc interval was also prolonged in the *cFat10*^*−/−*^ hearts, suggesting abnormal repolarization, which may be partly attributed to the increased late current in the model. *I*_NaL_ has been linked to the manifestation of inherited and acquired cardiac diseases, including long QT variant 3 syndrome and heart failure^36,37^.

The *cFat10*^*−/–*^ mice exhibited abnormal *I*_Na_ and ECG parameters, but did not show ventricular arrhythmias at baseline and developed ventricular arrhythmia after MI. Changes in *I*_Na_ observed at baseline in the *cFat10*^*−/−*^ hearts were modest and likely insufficient to provoke spontaneous ventricular arrhythmias. Thus, additional electrical and/or structural perturbations, such as ischemic events, were needed to unmask ventricular arrhythmias. The secondary changes included alterations in peak *I*_Na_ and *I*_NaL_ as well as reduced conduction velocity, prolonged ventricular AP, and promotion of early afterdepolarizations, which could lead to a predisposition to the reentrant circuit and trigger activity^33^.

Our findings on the regulatory effects of FAT10 on sodium channels might shed light on the role of UBLs in ischemia-induced arrhythmia, providing additional insight into the complex mechanisms underlying arrhythmogenesis during MI. In addition, FAT10 affects the expression of Cav3, a critical scaffolding protein involved in the genesis of t-tubules and the t-tubular Ca^2+^ current. We speculate that FAT10 may have similar modulatory effects on cardiac electrical activity in other pathophysiological conditions, such as heart failure and hypertrophic cardiomyopathy, which needs to be further investigated.

Several limitations should be considered in this study. First, because our experiments were confined to young adult mice, the findings might not pertain to older mice, which may exhibit arrhythmias secondary to age-associated changes, such as fibrosis^38^. Second, other Ca^2+^ or K^+^ channel proteins were not detected in the tandem mass tag-mass spectrometry proteomics experiments, which might be due to the limitation of the methods. Therefore, the potential role of Ca^2+^ and K^+^ currents, which are also crucial in AP repolarization, was not investigated. However, WB showed that Cav1.2 expression was not significantly changed in *cFat10*^*−/−*^ cells, which might exclude the effect of Ca^2+^ channels. Third, the protective role of FAT10 in ischemic arrhythmia was not tested in a cardiac FAT10 overexpression mouse model. Finally, the study was mainly focused on the susceptibility of *cFat10*^*−/−*^ mice to arrhythmias under ischemia. However, modulatory effects on cardiac electrical activity in other pathophysiological conditions (e.g., aging and hypertrophic cardiomyopathy) remain unexplored.

In conclusion, these findings provide the first evidence of a protective role of FAT10 in ischemia-induced ventricular arrhythmia. Deletion of the *Fat10* gene was associated with abnormal APD because of a dysregulated sodium current as a mechanism for ischemia-induced ventricular arrhythmia. In addition, the removal of *Fat10* was related to the reduced Nav1.5/FAT10 binding and increased Neddylation of Nav1.5, resulting in its subsequent degradation reduced expression levels and thereby explaining the molecular mechanisms underlying the abnormal sodium current. Our findings highlight the significance of FAT10 in regulating ventricular arrhythmia during MI.

## Materials and methods

### Cardiac *Fat10* knockout mouse model and generation of CRISPR-Cas9 knockout HEK293 cell lines

Cardiac conditional knockout of the *Fat10* gene in C57B/6L mice was based on homologous recombination technology with help from the Model Animal Research Center of Nanjing University. Briefly, when the *Fat10* gene is located between two loxP sites, the Cre enzyme is expressed, loxp can be deleted, and a frameshift mutation occurs. Subsequent knockdown of *Fat10* was further confirmed by WB and DNA-sequencing. *Fat10* deletion was induced by tamoxifen 6 weeks after the mice were born. The generation of *CRISPR-Cas9* knockout HEK293 cell lines was previously reported^14^. In brief, HEK293 cells were transfected with *CRISPR-Cas9* plasmids for 2 days and then treated with 1 µg/ml puromycin to enrich for transfected cells. Then, the viable clones were picked up, and *Fat10* knockout HEK293 cells were confirmed by both WB and DNA-sequencing.

### Animal and ischemic animal model

Newborn (6–10 g, 2 days) male Sprague–Dawley (SD) rats and C57/B6L mice (20–25 g, 12–16 weeks) were used in this study. All animal experiments and procedures were approved by the Animal Ethics Committee of Nanchang University and performed in accordance with the Guide for the Care and Use of Laboratory Animals.

As previously described^15^, adult C57/B6L mice were randomly used to create an MI model by ligating the left anterior descending coronary artery of the heart.

### Cell lines and cell culture

NRCMs were isolated from the ventricular myocardia of newborn SD rats through an enzymatic digestion method according to previous studies^15,39^. Cultured cardiomyocytes were subjected to hypoxic injury by incubation in a CO_2_/N_2_/O_2_ (94:5:1 ratio) environment at 37 °C for 8 h.

HEK293 cells were purchased from American Type Culture Collection (Rockville, USA) and cultured in Dulbecco’s modified Eagle’s medium containing 10% fetal bovine serum. Cells were cultured at 37 °C and 5% CO_2_ incubator in a humidified atmosphere.

### Cellular electrophysiology

The APs were recorded by whole-cell patch-clamp experiments in the current-clamp mode as previously described^39^. For recording, the isolated ventricular myocytes were kept at room temperature and perfused for 5 min with Tyrode’s solution^39^. The APs were stimulated with 5 ms, 900 mV square-wave pulses of 2 Hz pacing frequencies. All patch-clamp data were recorded using an EPC-10 patch-clamp amplifier (HEKA Electronik, Lambrecht, Germany).

The conventional whole-cell patch-clamp technique was used to record the Na^+^ current from HEK293 cells and rod-shaped, quiescent singly isolated mouse ventricular myocytes. The sodium current of HEK293 cells was recorded using a previously described method and standard solutions^40^.

### Liquid chromatography with tandem mass spectrometry (LC-MS/MS) analyses

LC-MS/MS analyses were performed as previously described^41^. Briefly, proteins were extracted from three *cFat10*^*−/−*^ and control mouse hearts and analyzed by label-free tandem mass tag and LC-MS/MS. The obtained samples were then analyzed by LC-MS/MS with the help of Shanghai Applied Protein Technology Co., Ltd., who provided technological assistance. The MS data were analyzed using MaxQuant software version 1.3.05 (Max Planck Institute of Biochemistry in Martinsried, Germany).

### ECG recordings, echocardiography, IF, histology, and immunohistochemical staining

ECG, echocardiography, IF, histology, and immunohistochemical staining were performed as previously described^15,39^.

### Plasmid and adenovirus constructs

The Flag-*Scn5a*, deletion forms of Nav1.5 (1–131, 132–415, 416–711, 712–939, 940–1200, 1201–1470, 1471–1523, 1524–1722, and 1773–2016 amino acids (a.a.)) and 1773–2016 a.a. mutant forms (SCN5A-C-ter-Mut) (lysine mutated to arginine) were purchased from GeneChem (Shanghai Co., Ltd). In addition, HA-tagged *Fat10* and His-tagged *Nedd4-2* cDNA were synthesized and subcloned into a recombinant adenovirus shuttle vector (pLenti6.3_MCS_IRES2). All of these recombinant adenovirus vectors were then transfected into packaged cells (Invitrogen, USA) for the generation of recombinant adenoviruses.

### qRT-PCR, Co-IP, WB, 2, 3, 5-triphenyltetrazolium chloride (TTC) staining, in vitro ubiquitination and GST pull-down assays

qRT-PCR Co-IP, WB, TTC staining, in vitro ubiquitination, and GST pull-down assays were performed as previously described^15,39^. Purified GST, GST-*Scn5a* fusion proteins, and His-FAT10 (CST, Cat#: Ag27712) were incubated with Glutathione Sepharose 4B beads (Sigma, USA) and analyzed by WB. The following primary antibodies were used: tubulin (1:1000; Proteintech, Cat#: 66031-1-Ig) and Na^+^/K^+^ ATPase (1:1000; Alomone Labs, Cat#: ANP-001). The Na^+^/Ca^2+^ exchanger (1:1000; Thermo, Cat#: MA3-926), Nav1.5 (1:200; Alomone Labs, Cat#: ASC-005), Nedd4-2 (1:1000; CST, Cat#: 4013), Connexin-43 (1:1000; Abcam, Cat#: ab11370), FAT10 (1:500; Abcam, Cat#: ab168680), Flag (1:1000; Proteintech, Cat#: 66008-3-Ig), His (1:1000; Proteintech, Cat#: 66005-1-Ig), HA (1:1000; Proteintech, Cat#: 51064-2-AP), GST (1:1000; Proteintech, Cat#:10000-0-AP), ubiquitin (1:1000; Abcam, Cat#: ab7780), SAP97 (1:1000; Thermo, Cat#: PA1-741), and MOG1 (1:500; Abcam, Cat#: ab15706) antibodies were used.

### Statistical analysis

Experiments were independently repeated a minimum of three times. Continuous data are presented as the mean ± standard error of the mean. Unpaired Student’s *t* tests (equal variance) or unpaired Student’s *t* tests with Welch’s correction (unequal variance) were used for comparisons between two independent groups, and one-way analysis of variance with Tukey’s post hoc test was used for multiple comparisons. Categorical data are expressed as percentages and were compared between groups using Fisher’s exact test. All analyses were performed using the GraphPad Prism 5.0 software. Statistical significance was defined as *p* < 0.05.

## Supplementary information

Figure S1

Figure S2

Figure S3

Figure S4

Figure S5

Figure S6

Supplementary Figure Legends

Supplementary Tables
