# Supplementary figures and images for: FAT10 protects against ischemia-induced ventricular arrhythmia by decreasing Nedd4-2/Nav1.5 complex formation
Source: Cell Death Dis. 2021 Jan 5;12(1):25. doi: 10.1038/s41419-020-03290-3 (PMC7790828; doi:10.1038/s41419-020-03290-3)

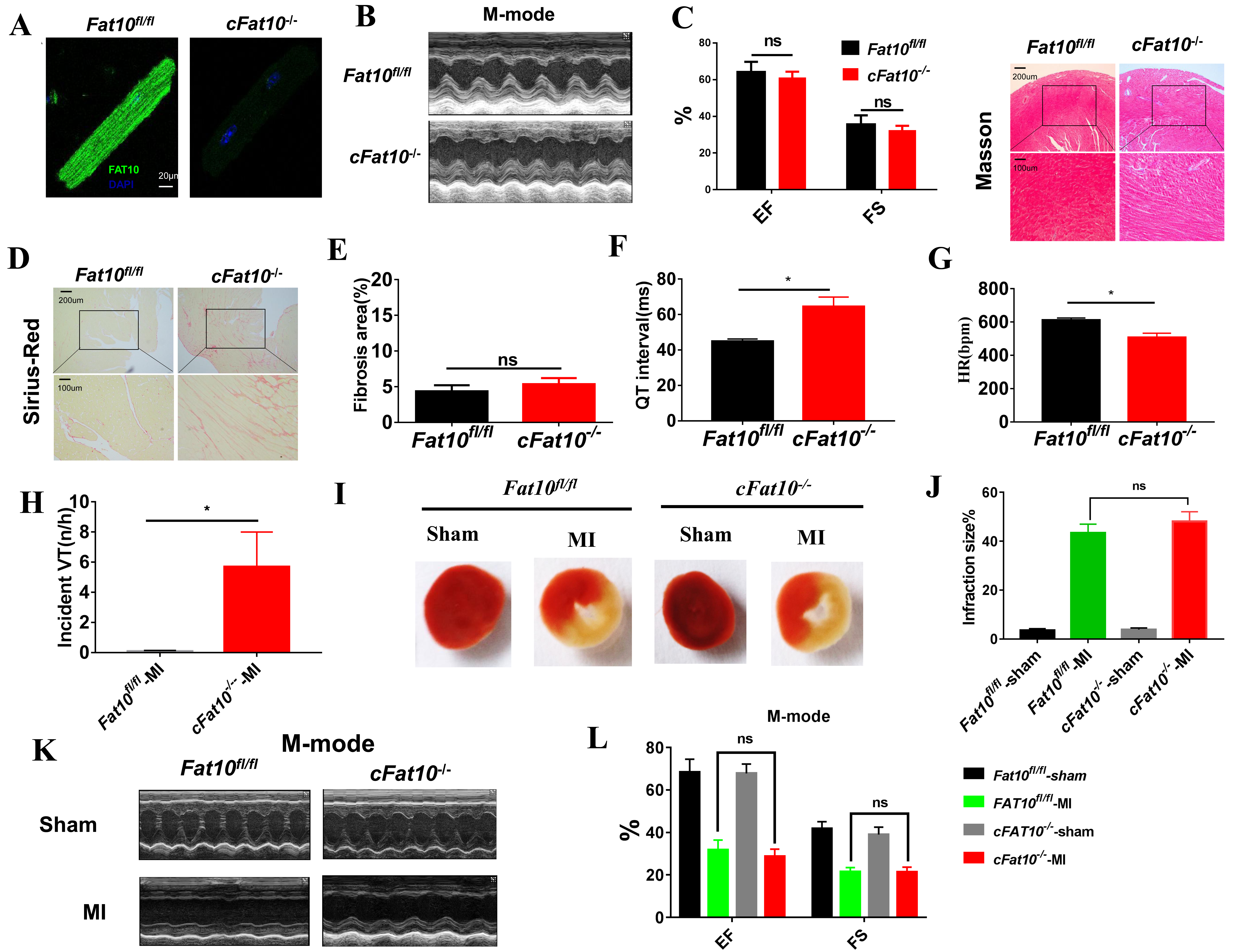

Supplement: Supplementary file 1 — Figure S1 [file 41419_2020_3290_MOESM1_ESM.tif]

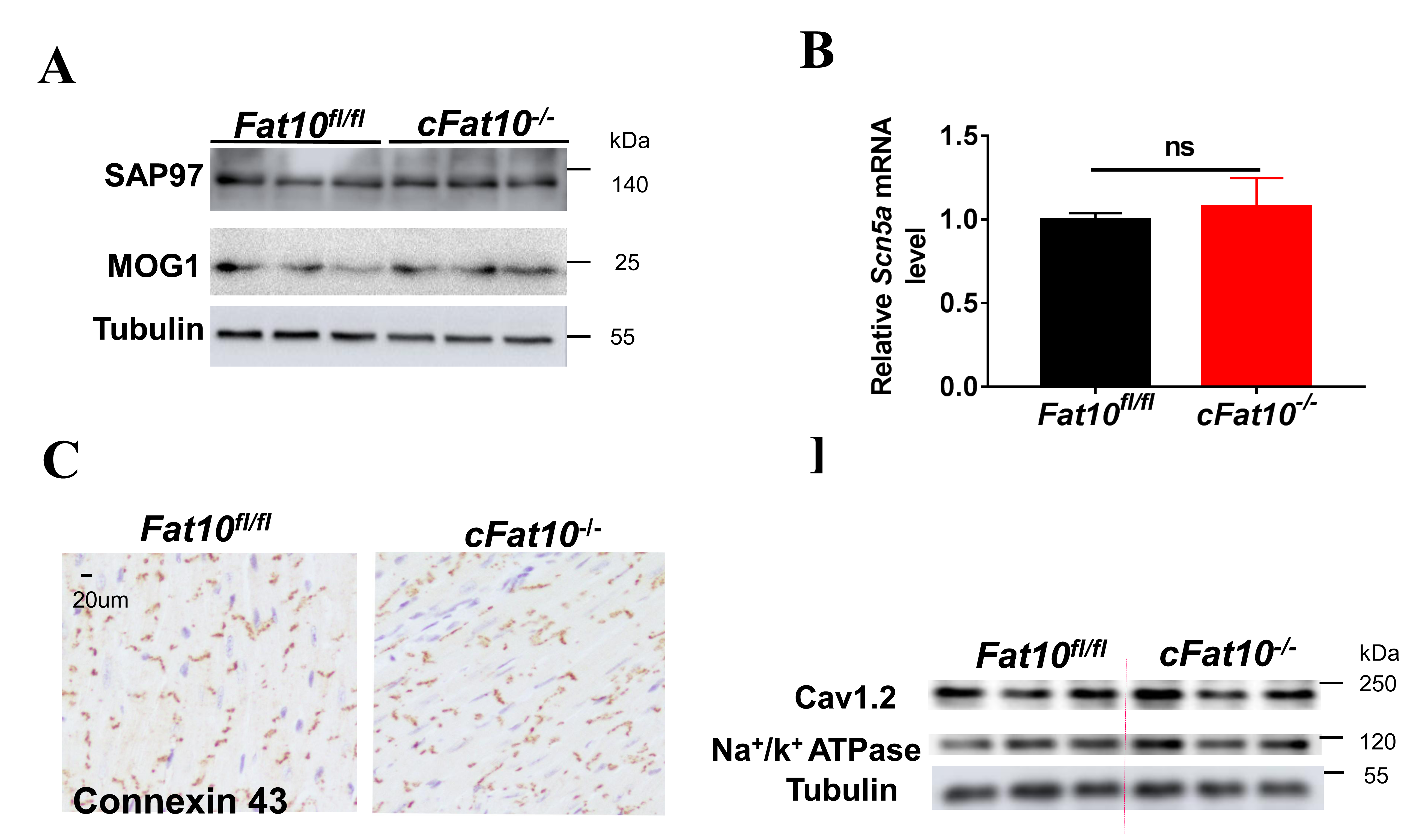

Supplement: Supplementary file 2 — Figure S2 [file 41419_2020_3290_MOESM2_ESM.tif]

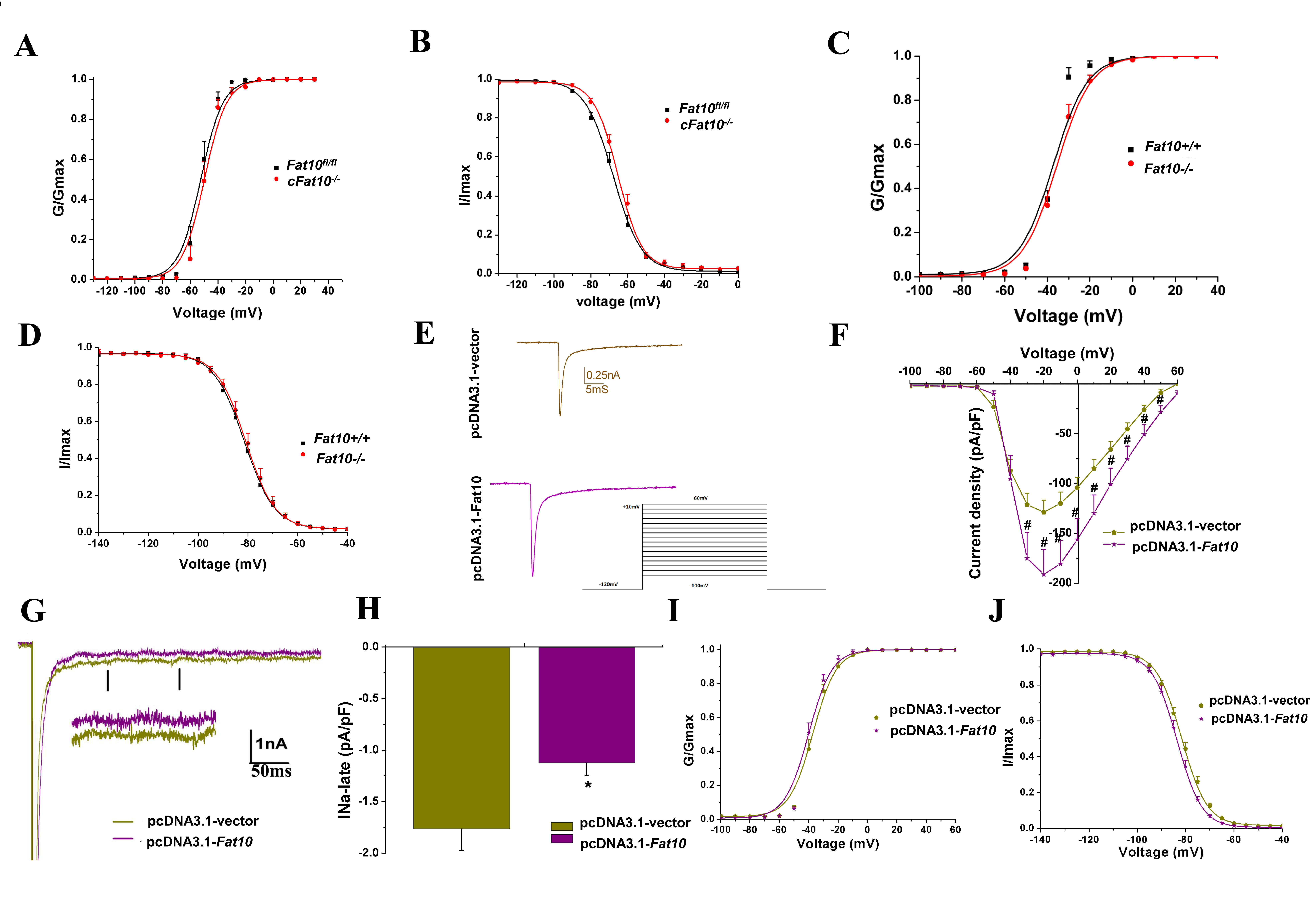

Supplement: Supplementary file 3 — Figure S3 [file 41419_2020_3290_MOESM3_ESM.tif]

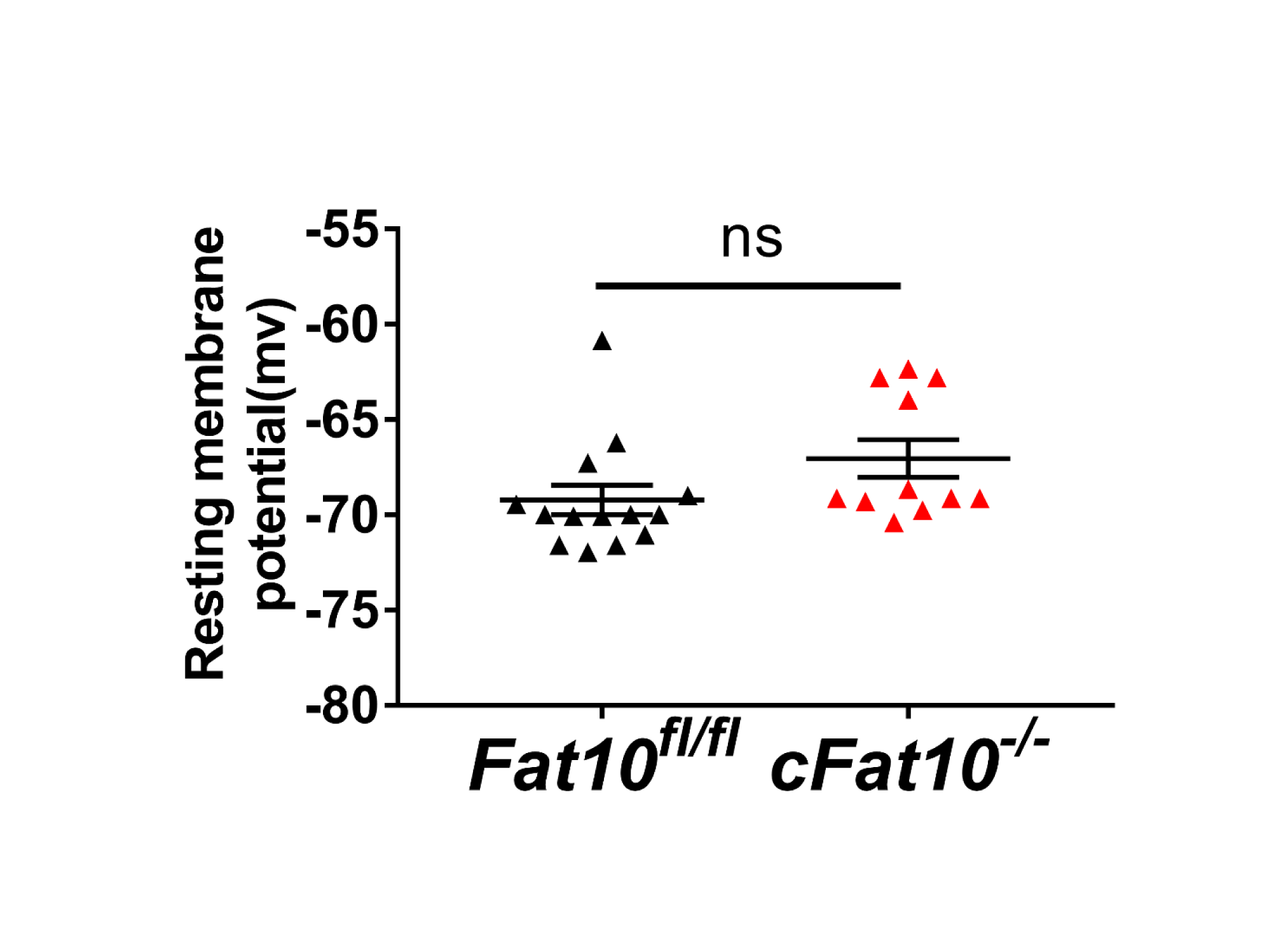

Supplement: Supplementary file 4 — Figure S4 [file 41419_2020_3290_MOESM4_ESM.tif]

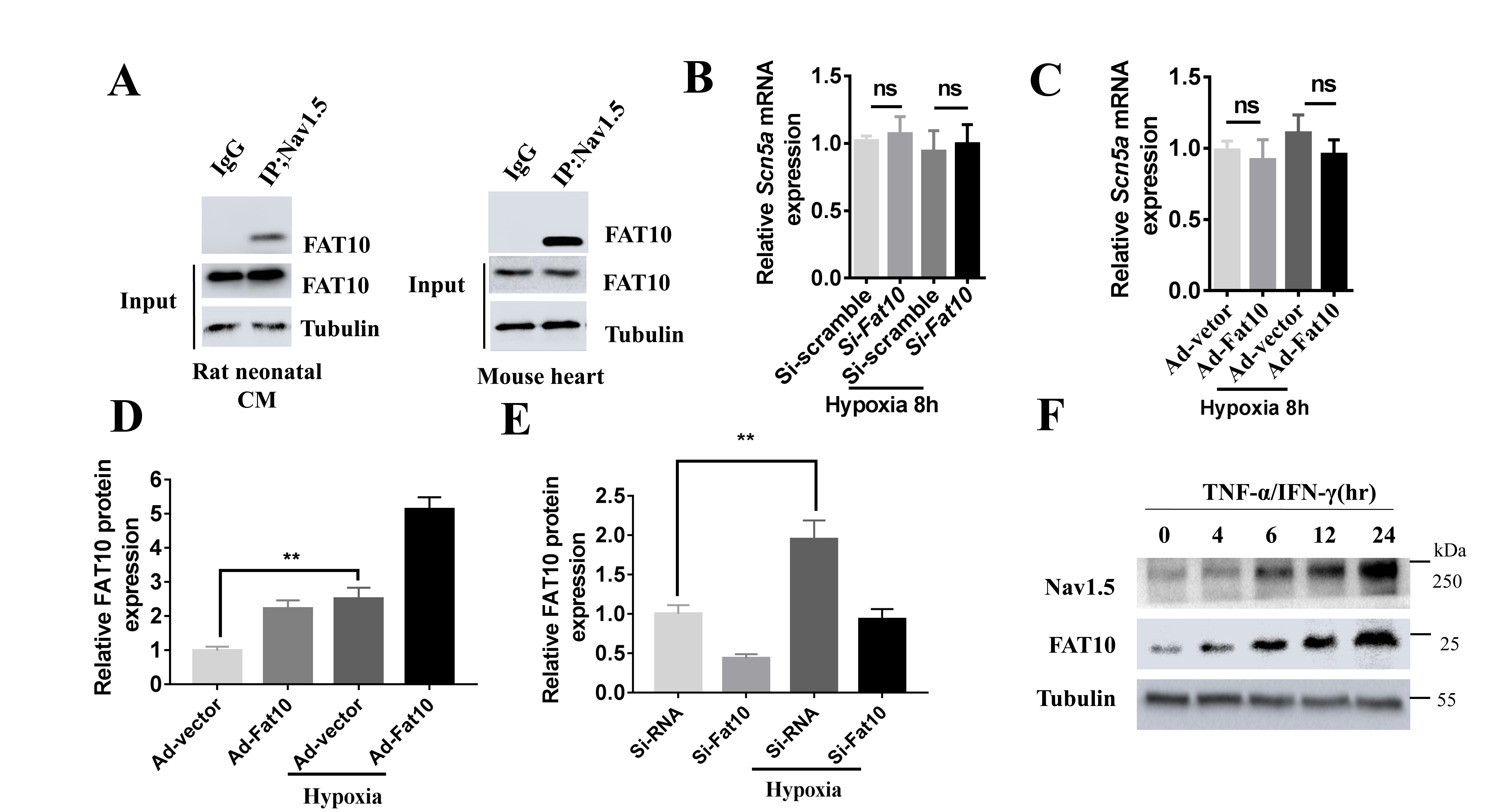

Supplement: Supplementary file 5 — Figure S5 [file 41419_2020_3290_MOESM5_ESM.tif]

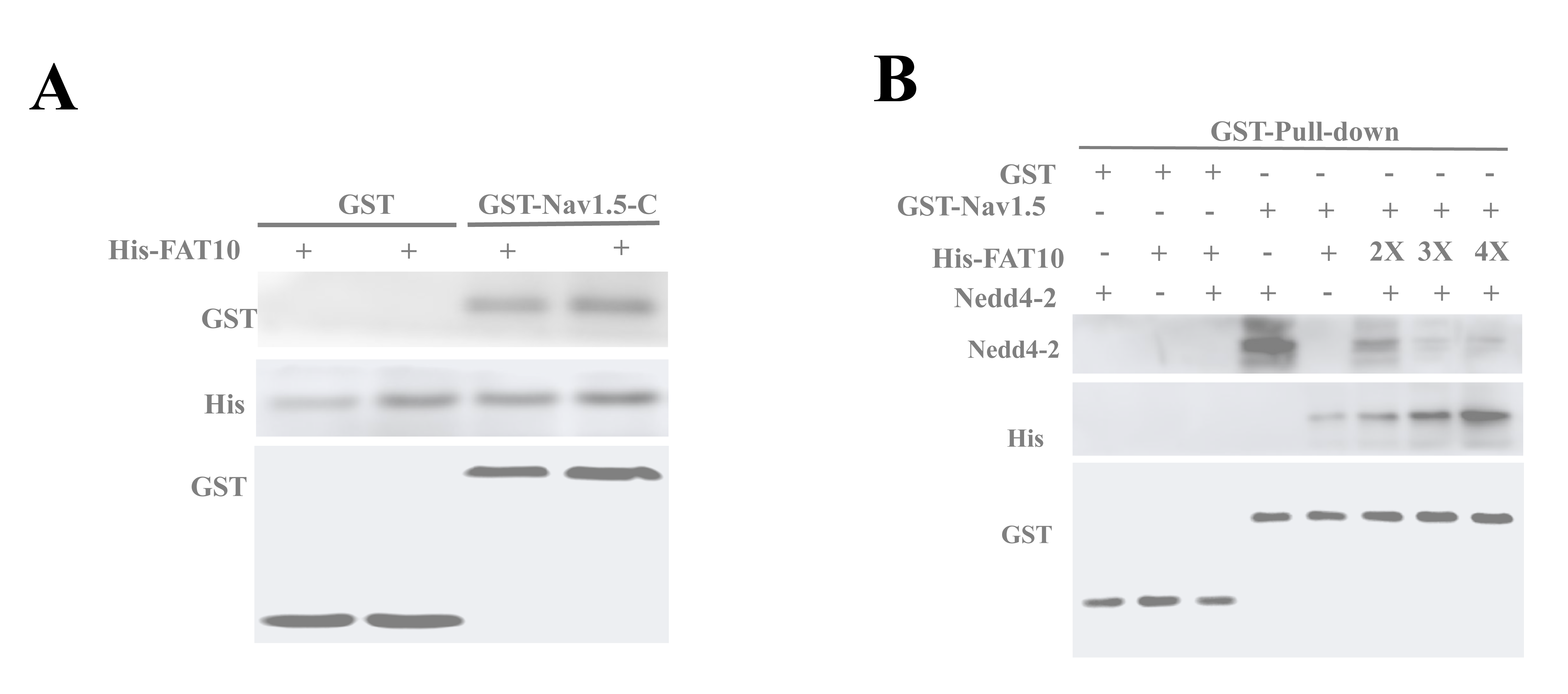

Supplement: Supplementary file 6 — Figure S6 [file 41419_2020_3290_MOESM6_ESM.tif]
