# Supplementary material for: FAT10 protects against ischemia-induced ventricular arrhythmia by decreasing Nedd4-2/Nav1.5 complex formation
Source: Cell Death Dis. 2021 Jan 5;12(1):25. doi: 10.1038/s41419-020-03290-3 (PMC7790828; doi:10.1038/s41419-020-03290-3)
Supplement: Supplementary file 7 — Supplementary Figure Legends [file 41419_2020_3290_MOESM7_ESM.docx]

**FAT10 protects against ischemia-induced ventricular arrhythmia by decreasing Nedd4-2/Nav1.5 complex formation**

Xiao Liu1*, M.D., Ph.D.; Jin Ge1*, M.D. ; Chen Chen1*, M.D. ; Yang Shen2, M.D., Ph.D. ; Jinyan Xie2, M.S. Xin Zhu1, M.D. ; Menglu Liu1, M.D. ; Jinzhu Hu1, M.D. , Ph.D.; Leifeng Chen2, M.D. , Ph.D.; Linjuan Guo1, M.D. ; Qiongqiong Zhou1, M.D.,Ph.D. ; Xia Yan2, B.S. ; Yuming Qiu3, M.D. ; Rong Wan#2, Ph.D., Ali J. Marian4, M.D., Kui Hong#1,2, M.D., Ph.D.

# Supplemental Figures legends

# Figure S1. Effect of *Fat10*-deletion on cardiac structure and electrocardiograph parameters.

**a.** Representative photomicrographs showing immunostaining for FAT10 in cardiomyocytes isolated from *Fat10^fl/fl^* and *cFat10^-/-^* mice. **b.** Representative example (left) or quantitative data (right) of M-mode echocardiograms of *Fat10^fl/fl^* and *cFat10^-/-^* mice (EF: ejection fraction, FS: [shortening](javascript:;) [fraction](javascript:;), ns: no significance). **c, d.** Masson staining **(c)**, Sirius red staining **(d)** images of heart sections from *Fat10^fl/fl^* and *cFat10^-/-^* mice. **e.** Average data of cardiac fibrosis in *Fat10^fl/fl^* and *cFat10^-/-^* mice. **f, g.** Analysis of electrocardiograph parameters in freely moving *cFat10^-/-^* mice (n=6) and *Fat10^fl/fl^* (n=10) littermate controls. QT interval **(f)**, and HR **(g)** (**p*<0.05). **h.** Average incident of ventricular tachycardia (VT) incident in *cFat10^-/-^* and *Fat10^fl/fl^* (n=10) littermate controls after myocardial infraction (**p*<0.05). **i-j.** TTC staining showing infraction size in *cFat10^-/-^* and *Fat10^fl/fl^* mice hearts 3 days after LAD ligation **(i)** and the average data **(j). k-l.** Typical example of M-mode echocardiograms**(k)** and cardiac function**(l)** of *cFat10^-/-^* and *Fat10^fl/fl^* mice hearts with myocardial infraction or without myocardial infraction (sham); N = 7 mice for each group; (EF: ejection fraction, FS: [shortening](javascript:;) [fraction](javascript:;), ns: no significance).

# Figure S2: Effect of cardiomyocyte *Fat10*-deletion on connexin-43 and key proteins mediating Nav1.5 trafficking.

**a.** Western blot was performed to detect the expression of Nav1.5 trafficking protein SAP-97, and MOG1 protein levels in heart tissues from *cFat10^-/-^* and *Fat10^fl/fl^* mice. **b.** Quantitative real-time PCR analysis was performed to detect the mRNA level of *Scn5a* in heart tissue samples from *cFat10^-/-^* and *Fat10^fl/fl^* mice (ns, no significance). **c.** Immunohistochemistry staining showing the expression of connexin 43 in heart tissues from *cFat10^-/-^* and *Fat10^fl/fl^* mice. **d.** Western blot showing the expression of Cav1.2 and Na^+^/K^+^ ATPase in heart tissues from *cFat10^-/-^* and *Fat10^fl/fl^* mice**.** All results are expressed as mean±SEM of independent experiments.

# Figure S3: Steady-state activation and inactivation plots for *wild type*, mutant, and native Nav1.5 with FAT10 overexpression or inhibition.

**a, b.** Steady-state activation **(a)** and inactivation **(b)** of ventricular myocytes isolated from *cFat10^-/-^* (n=13) and *Fat10^fl/fl^* (n=15). **c, d.** Steady-state activation **(c)** and inactivation **(d)** of HEK293 (*Fat10^+/+^*) and *Fat10-KO* (*Fat10^-/-^*) HEK293 cells. **e, f.** Effect of FAT10 overexpression on I_Na_ in HEK293 cells expressing *wild type-Scn5a*. Representative traces of I_Na_ with a pulse protocol **(e)** and the current–voltage relationship of I_Na_ in HEK293 cells transfected with *vector plasmid (pcDNA3.1-vector)* (cells=19) or *Flag-Fat10* plasmid *(pcDNA3.1-Fat10)* (cells=7) in HEK293 cells (F) (#*p*<0.05, *pcDNA3.1-Fat10 vs. pcDNA3.1-vector*). **g, h.** I_Na,L_ was measured in response to 150 ms voltage steps to −20 mV from a holding potential of −120 Mv (Mean I_Na_ at 150 ms, **p*<0.05, *pcDNA3.1-Fat10 vs. pcDNA3.1-vector*). **i, j.** Steady-state activation **(i)** and inactivation **(j)** in HEK293 cells transfected with *vector plasmid* or *Flag-Fat10* plasmid. All results are expressed as mean±SEM of independent experiments.

**Figure S4:** **Effect of cardiomyocyte *Fat10*-deletion on** **resting membrane potential.**

Resting membrane potential were detected in cardiomyocytes isolated from *cFat10^-/-^* and *Fat10^fl/fl^* mice (n = 14 cells for *Fat10^fl/fl^* and n = 11 cells for *cFat10^-/-^*) (ns, no significance).

# Figure S5: Effect of FAT10 on Nav1.5 protein and mRNA expression in cardiomyocytes.

**a.** Co-immunoprecipitation (Co-IP) was performed to detect the connection between FAT10 and Nav1.5 in rat neonatal cardiomyocytes (left) and mouse hearts (right). **b, c.** Effect of FAT10 knockdown **(b)** or FAT10 overexpression **(c)** on *Scn5a* mRNA expression in rat neonatal cardiac myocytes under normoxic and hypoxic conditions. **d.** **e.** Quantitative analysis of effect of FAT10 knockdown **(d)** or FAT10 overexpression **(e)** on FAT10 expression in rat neonatal cardiac myocytes under normoxic and hypoxic conditions. **f.** Representative immunoblots of Nav1.5 levels in NRCMs treated with IFN-γ/TNF-α. Ns: non-significant; **p*<0.05; * * *p*<0.01. All results are expressed as mean±SEM of independent experiments.

# Figure S6: FAT10 directly interacts with Nav1.5 and regulates ubiquitination by competing with Nedd4-2.

**a.** Direct interaction of Nav1.5-C-terminus with FAT10 according to GST pull-down assay. Recombinant GST and GST-Nav1.5 were incubated with recombinant His-FAT10 and GSH beads. **b.** Competitive binding of Nav1.5 was analyzed in a GST pull-down experiment. Recombinant GST and GST-Nav1.5 were incubated with recombinant His-FAT10 and GSH beads. All results are expressed as mean±SEM of independent experiments.
