# Supplementary material for: FAT10 protects against ischemia-induced ventricular arrhythmia by decreasing Nedd4-2/Nav1.5 complex formation
Source: Cell Death Dis. 2021 Jan 5;12(1):25. doi: 10.1038/s41419-020-03290-3 (PMC7790828; doi:10.1038/s41419-020-03290-3)
Supplement: Supplementary file 8 — Supplementary Tables [file 41419_2020_3290_MOESM8_ESM.docx]

**FAT10 protects against ischemia-induced ventricular arrhythmia by decreasing Nedd4-2/Nav1.5 complex formation**

Xiao Liu1*, M.D., Ph.D.; Jin Ge1*, M.D. ; Chen Chen1*, M.D. ; Yang Shen2, M.D., Ph.D. ; Jinyan Xie2, M.S. Xin Zhu1, M.D. ; Menglu Liu1, M.D. ; Jinzhu Hu1, M.D. , Ph.D.; Leifeng Chen2, M.D. , Ph.D.; Linjuan Guo1, M.D. ; Qiongqiong Zhou1, M.D.,Ph.D. ; Xia Yan2, B.S. ; Yuming Qiu3, M.D. ; Rong Wan#2, Ph.D., Ali J. Marian4, M.D., Kui Hong#1,2, M.D., Ph.D.

**Supplemental tables**

**Table S1.** Cardiac function measured by echocardiography

| Cardiac function | *Fat10^fl/fl^* (n=12) | *cFat10^-/-^*(n=11) | P |
| --- | --- | --- | --- |
| LVVol;d | 37.36±0.69 | 39.75±1.18 | 0.08 |
| LVVol;s | 13.36±0.98 | 13.43±0.60 | 0.36 |
| IVSd(mm) | 0.78±0.08 | 0.67±0.01 | 0.08 |
| IVSs(mm) | 0.94±0.36 | 0.84±0.02 | 0.36 |
| LVIDd(mm) | 3.02±0.64 | 3.12±0.03 | 0.64 |
| LVIDs(mm) | 1.84±0.56 | 1.99±0.03 | 0.55 |
| LVPWd(mm) | 0.98±0.14 | 0.77±0.02 | 0.14 |
| LVPWs(mm) | 1.24±0.08 | 0.92±0.03 | 0.07 |
| LVVold: left ventricular volume at diastolic phase; LVVols: left ventricular volume at systolic phase ; SV: stroke volume; LVIDd: left ventricular internal diameter at diastolic phase; LVIDs: left ventricular internal diameter at systolic phase; IVSd: interventricular septal thickness at diastolic phase; IVSs: interventricular septal thickness at systolic phase; LVPWd: left ventricular posterior wall thickness at diastolic phase; LVPWs: left ventricular posterior wall thickness at systolic phase. | | | |
